# Impact of ICD-10-CM Transition on Mental Health Diagnoses Recording

**DOI:** 10.5334/egems.281

**Published:** 2019-04-12

**Authors:** Christine C. Stewart, Christine Y. Lu, Tae K. Yoon, Karen J. Coleman, Phillip M. Crawford, Matthew D. Lakoma, Gregory E. Simon

**Affiliations:** 1 Kaiser Permanente Washington, US; 2 Harvard Medical School, US; 3 Kaiser Permanente Southern California, US; 4 Kaiser Permanente Northwest, US

**Keywords:** mental health, ICD-10-CM, depression, psychosis, bipolar disorder, electronic health record

## Abstract

**Objective:**

This study examines the impact of the transition from ICD-9-CM to ICD-10-CM diagnosis coding on the recording of mental health disorders in electronic health records (EHRs) and claims data in ten large health systems. We present rates of these diagnoses across two years spanning the October 2015 transition.

**Methods:**

Mental health diagnoses were identified from claims and EHR data at ten health care systems in the Mental Health Research Network (MHRN). Corresponding ICD-9-CM and ICD-10-CM codes were compiled and monthly rates of people receiving these diagnoses were calculated for one year before and after the coding transition.

**Results:**

For seven of eight diagnostic categories, monthly rates were comparable during the year before and the year after the ICD-10-CM transition. In the remaining category, psychosis excluding schizophrenia spectrum disorders, aggregate monthly rates of decreased markedly with the ICD-10-CM transition, from 48 to 33 per 100,000. We propose that the change is due to features of General Equivalence Mappings (GEMS) embedded in the EHR.

**Conclusions:**

For most mental health conditions, the transition to ICD-10-CM appears to have had minimal impact. The decrease seen for psychosis diagnoses in these health systems is likely due to changes associated with EHR implementation of ICD-10-CM coding rather than an actual change in disease prevalence. It is important to consider the impact of the ICD-10-CM transition for all diagnostic criteria used in research studies, quality measurement, and financial analysis during this interval.

## Introduction

Although the International Classification of Diseases Ninth Revision, Clinical Modification (ICD-9-CM) was developed for systematic study of illness, it is an essential component of the structured language of health care financing and evaluation of medical care. Electronic health records (EHRs), incentivized by the Health Information Technology for Economic and Clinical Health (HITECH) Act of 2009, significantly changed the process for collecting diagnosis data, eliminating paper and shifting the workflows of providers and coders [1,2]. Most clinical EHR systems now provide clinical terminology mapping, which allow clinicians to choose from a list of options displayed in response to the text they enter. Given the gradual adoption of EHR and the development of clinical terminology tools, these changes have had minimal disruption for the myriad uses of diagnosis data at the population level, and in fact EHR data has been very beneficial for epidemiological research [3]. For example, the contribution of Hepatitis C to mortality was recognized only after the widespread adoption of electronic diagnosis records [4].

In contrast, the recent adoption of International Classification of Diseases Tenth Revision, Clinical Modification (ICD-10-CM) coding in the United States occurred nationwide on October 1, 2015. ICD-10-CM represents a major revision, designed to better support the role of coding in reimbursement, quality measurement and monitoring [5]. Its potential disruption of the functioning of clinical delivery systems has long been recognized [6,7] and in fact resulted in several delays in its final adoption [8]. Major issues affecting health care operations were likely sorted out early in the ICD-10 era (for example, the failure of clinical terminology tools to suggest a reimbursable option or not excluding remission codes from a drug treatment denominator would become apparent to a health system very quickly). Subtler effects on revenue, such as risk-adjustment algorithms relying on a wide range of diagnoses, or on longer-term surveillance and population health would be expected to emerge more gradually.

An abrupt shift in coding or terminology mapping would be more problematic for activities dependent on a stable longitudinal relationship between diagnostic codes and clinical reality. That stability is essential for public health surveillance, health care quality metrics, and evaluating the impact of care improvement interventions. For example, amid increasing opioid-related mortality and federal and state efforts to halt and reverse the trend, ICD-10-CM expanded the number of relevant codes almost 5-fold. Analysis of opioid-related hospitalizations, which increased 5 percent and 3.5 percent per quarter before and after the ICD-10-CM, respectively, increased 14.1 percent between the last quarter coded in ICD-9-CM and the first quarter coded in ICD-9-CM [9]; while coding of adverse effects associated with therapeutic use increased, use of abuse and poisoning codes declined. For diabetes and hypertension, two chronic conditions for which care quality is commonly monitored, some ICD-10-CM definitions resulted in changes in diabetes diagnosis rates but hypertension rates remained steady [10]. We have previously used ICD-9-CM codes to identify first-episode psychosis [11] and to estimate the requirements for scaling successful early intervention programs [12]. Finally, self-inflicted injury and poisoning coding changed from requiring two codes, one for injury or poisoning and one for the external cause, to one code with the external cause embedded, which greatly increased the number of available codes. Accurately assessing rates of self-harm diagnoses is important in suicide prevention programs both as a risk factor and an early outcome; we previously reported differences before and after the ICD-10-CM transition [13]. The observed heterogeneity of the effect of the transition is not surprising given that translation of ICD-9-CM to ICD-10-CM codes ranges from simple to convoluted [3] depending on clinical category; substance-related and injury/poisoning are two of the more complicated chapters. Therefore, current recommendations include validating and adjusting any ICD-10-CM definitions that have not been previously used, and aggregating codes into groups where possible [7,9,10].

The Health Care Systems Research Network Virtual Data Warehouse (HCSRN VDW) [14] provides an efficient infrastructure for assessing the impact of coding changes across multiple organizations. Here we present a method and results from ten members of the HCSRN and Mental Health Research Network (MHRN) to identify and investigate the causes of discontinuities in diagnosis recording during this period. We examined several mental health disorder diagnosis groups, including depression, anxiety, attention deficit disorder, bipolar disorder, psychotic disorder, eating disorder and personality disorder, over a two-year period spanning the transition from ICD-9-CM to ICD-10-CM. We use these data to examine discontinuities in diagnosis rates related either to the coding transition itself or to changes in terminology mapping prompted by the coding change.

## Methods

Diagnoses were extracted from federated data warehouses containing electronic medical record and insurance claims data [14] from October 2014 through September 2016 at each of ten participating sites:

- HealthPartners (Minnesota)
- Harvard Pilgrim Health Care (Massachusetts)
- Henry Ford Health System (Michigan)
- Baylor, Scott & White Health (Texas)
- Six Kaiser Permanente regions
  - Colorado
  - Georgia
  - Hawaii
  - Southern California
  - Washington
  - Northwest

These health care systems serve an annual population of over 8 million members which reflects the demographic diversity of the associated geographic areas [15]. Responsible Institutional Review Boards at each health system approved waivers of consent for this research use of de-identified records data.

Definitions of mental health conditions for ICD-10-CM were developed using the ICD-9-CM definitions developed by the MHRN [16] and mapping tools from the Centers for Medicare and Medicaid Services [17]. These definitions are Available from https://github.com/MHResearchNetwork/Diagnosis-Codes. In each health system the monthly diagnosis rates for each class of 8 categories of diagnoses were calculated as the total number of enrolled members receiving at least one diagnosis in that category during that calendar month divided by the total number of members enrolled during that calendar month.

Where discontinuities were found, we stratified results by site and demographic variables. We also explored the source EHR data supporting the interface allowing clinicians to search for diagnosis codes. As with most EHRs, clinicians’ recording of encounter or billing diagnoses at these sites allowed either direct entry of diagnosis or entry of free text leading to suggested diagnosis codes. Each EHR database may include several commonly used free-text terms mapping to a single ICD-9-CM or ICD-10-CM diagnosis code.

## Results

**Figure 1:**
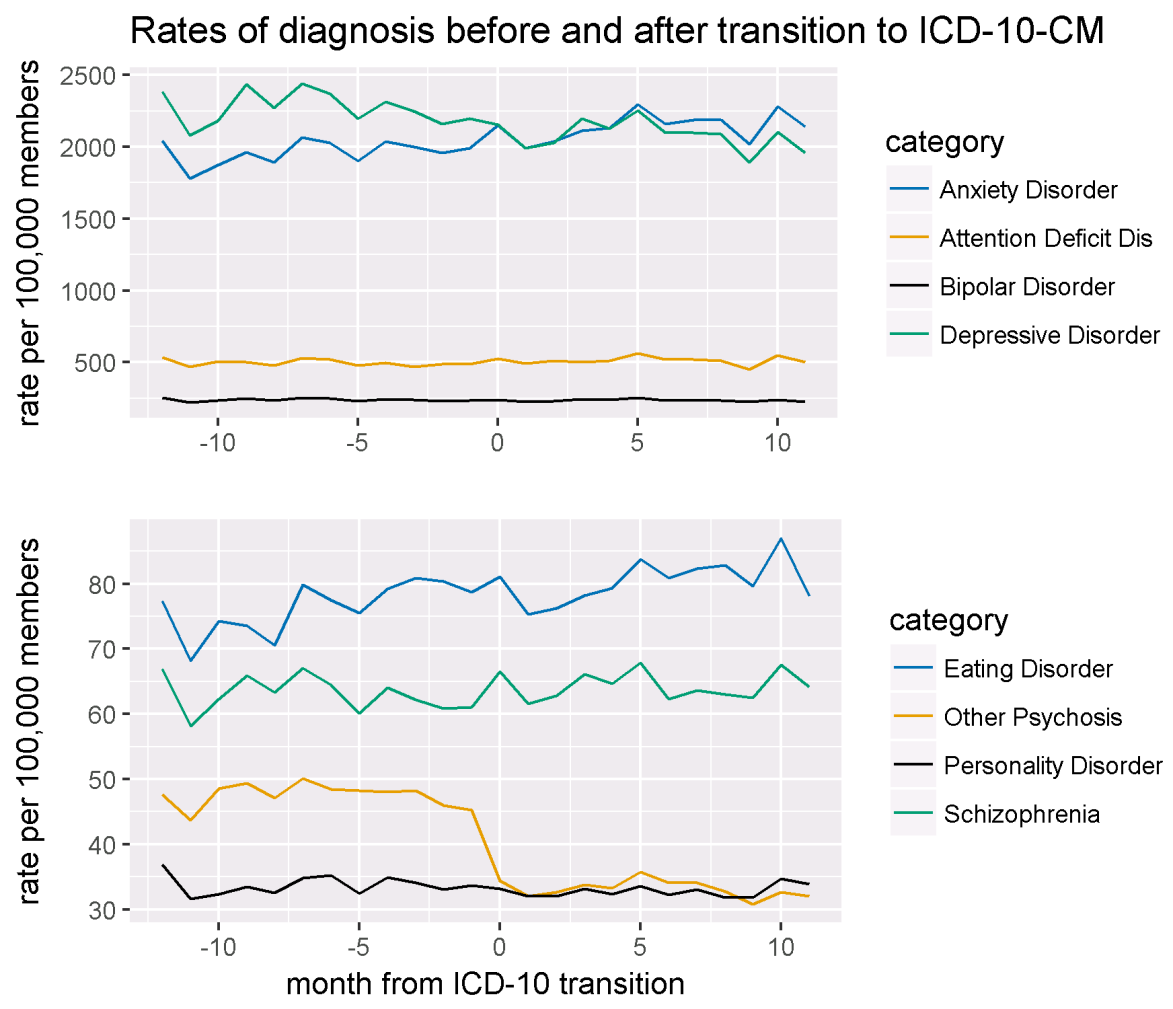
Aggregate rates of diagnosis of Schizophrenia and other psychosis before and after transition to ICD-10-CM (Oct. 2014–Sep. 2016).

Figure 1 shows monthly rates of eight classes of mental health diagnoses for all sites combined from October 1, 2014 to September 30, 2016. Monthly enrollment increased gradually during this period from 8.07 million to 9.01 million. Rates of bipolar disorder, attention deficit disorder, schizophrenia and personality disorder remained stable across the transition to ICD-10-CM. Anxiety and eating disorder rates show a small gradual increase over the entire two-year period, and depression rates decrease in a similarly gradual way. Site-stratified rates for these diagnosis categories are shown in Supplemental Figure 1.

**Figure 2:**
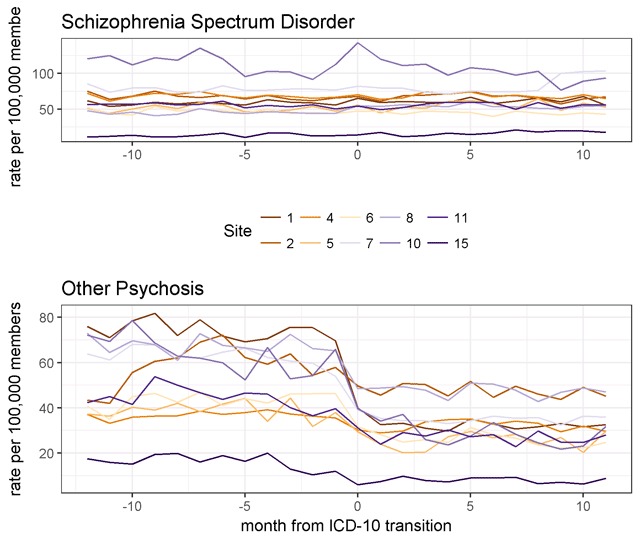
Rates of diagnosis of other psychosis at ten individual sites before and after transition to ICD-10-CM (Oct. 2014–Sep. 2016).

**Figure 3:**
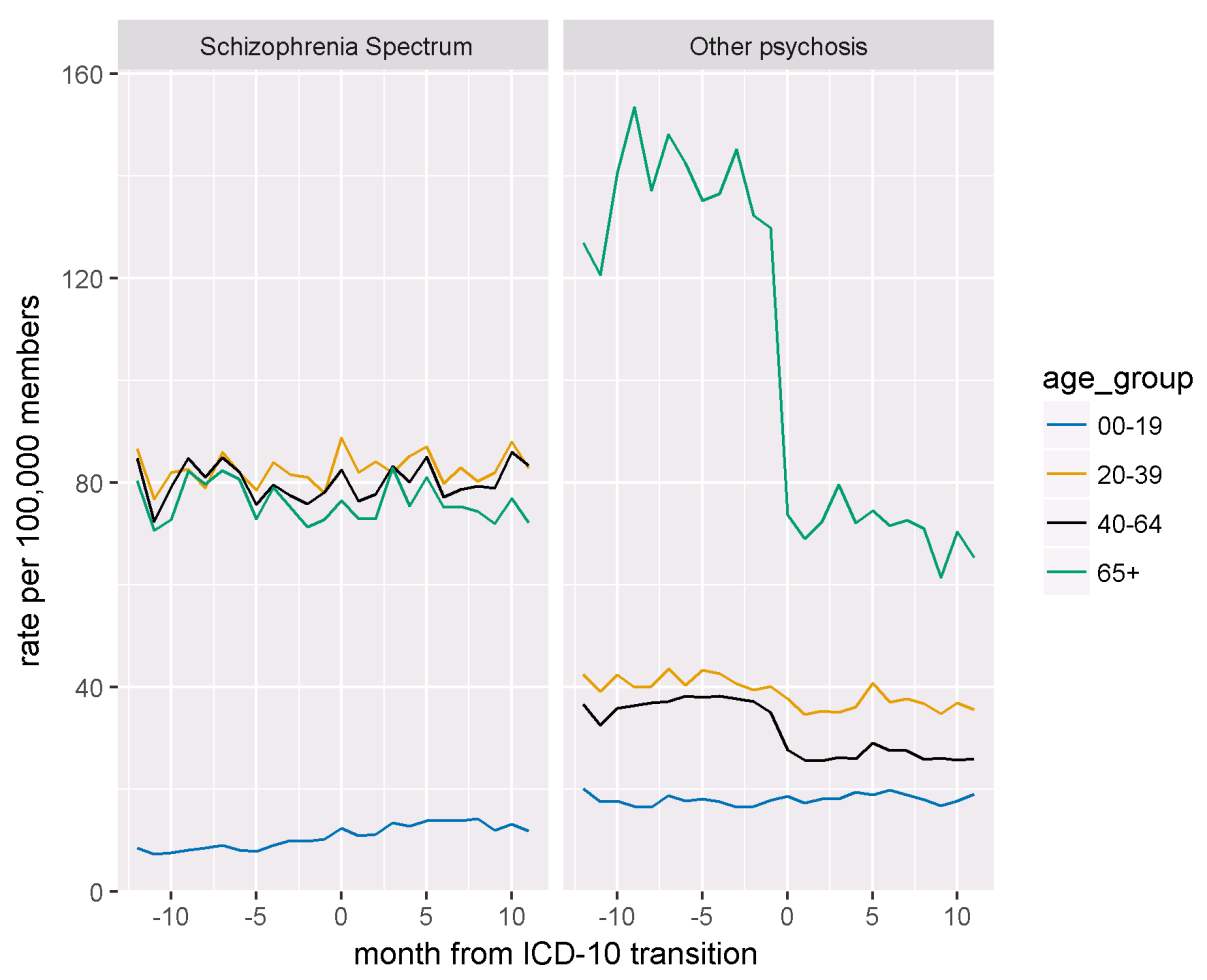
Age-stratified rates of Schizophrenia Spectrum and other psychotic disorders (Oct. 2014–Sep. 2016).

In contrast, the rate of psychotic disorder diagnoses other than schizophrenia decreased by almost 40 percent at the time of the transition to ICD-10-CM and remained relatively stable thereafter. Figure 2 shows time trends in schizophrenia spectrum and other psychotic disorder diagnoses for all ten health systems. While the rate of other psychotic disorder diagnoses decreased at all sites, proportional decreases ranged from approximately 30 percent at some sites to approximately 60 percent at others. Age-stratified analyses (Figure 3) revealed that members/patients aged 65 or older had both the highest rate of other psychotic disorder diagnoses and the largest decrease with the transition to ICD-10-CM.

Sites 1 and 4 were chosen for investigation of text associations with diagnosis codes in the EHR clinical interface because of a larger and smaller relative decrease in rate of other psychotic disorder diagnoses. As shown in Table 1, ICD-9-CM code 298.9 (Unspecified psychosis) was the most common code in this class (83 percent), and while the corresponding ICD-10-CM code (F29) was also the most common, it represented a smaller proportion of the category (59 percent).

**Table 1:** ICD-9-CM and ICD-10-CM codes for psychotic disorders and their frequency (Oct. 2016-Sep. 2016).

Schizophrenia Spectrum disorder	ICD-9-CM codes	N (%) of category	ICD-10-CM descriptions	ICD-10-CM codes	% of category

Schizophrenia	295–295.65, 295.8–295.95 (45 codes)	30319 (48.0)	Schizophrenia	F20-F20.9 (9 codes)	31708 (46.3)
Schizoaffective Disorder	295.7–295.75 (6 codes)	32813 (52.0)	Schizoaffective disorders	F25-F25.9 (4 codes)	36768 (53.7)
**Other Psychosis**					

Delusional disorder	297.1	5448 (11.4)	Delusional disorders	F22	10035 (28.4)
Shared psychotic disorder	297.3	5448 (0.2)	Shared psychotic disorder	F24	69 (0.2)
Schizotypal personality disorder	301.22	478 (1.0)	Schizotypal disorder	F21	581 (1.6)
Acute paranoid reaction Psychogenic paranoid psychosis Other and unspecified reactive psychosis	298.3 298.4 298.8	1806 (3.8)	Brief psychotic disorders	F23	2820 (8.0)
Excitative type psychosis	298.1	183 (0.4)	Other psychotic disorder not due to a substance or known physiological condition	F28	1009 (2.9)
Unspecified psychotic disorder not due to a substance or known physiological condition	298.9	39628 (83.2)	Unspecified psychosis not due to a substance or known physiological condition	F29	20763 (58.9)

**Figure 4:**
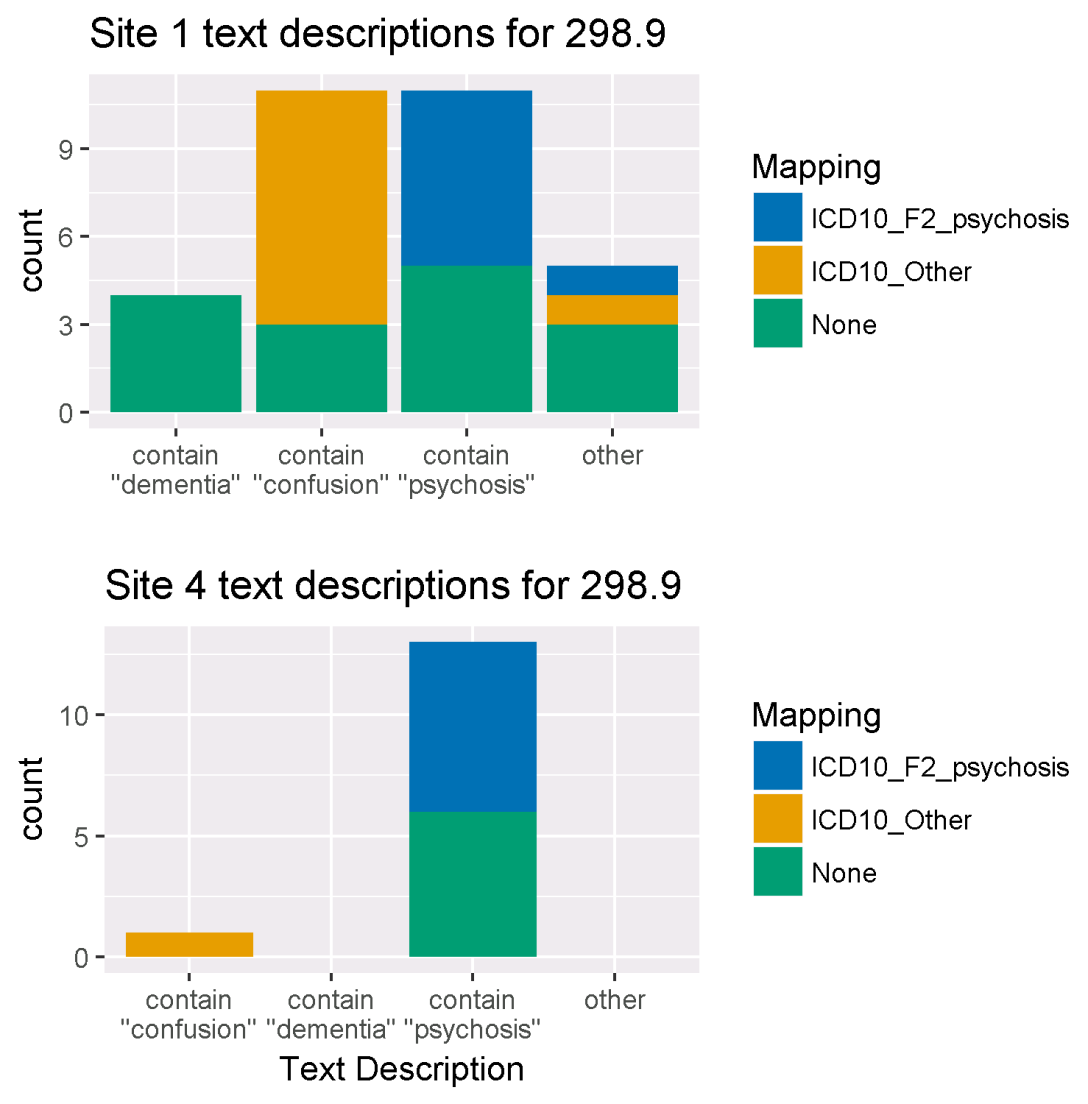
EHR text descriptions associated with ICD-9-CM 298.9 (Unspecified psychosis).

At site 1, ICD-9-CM code 298.9 was linked to 31 possible text descriptions. Of these 31 descriptions, eleven contained the word “psychosis”, eleven contained the word “confusion” and five contained the word “dementia.” Only 16 of these 31 terms were mapped to a corresponding ICD-10-CM code of F29 (Unspecified psychosis not due to a substance or known physiological condition). As shown in Figure 4A, terms containing the word “psychosis” were mapped to a code in the ICD-10-CM psychosis (F20–29) range, most often F29. In contrast, terms containing “confusion” or “dementia” were mapped to ICD-10-CM R41.0 (Disorientation), F44.89 (Other dissociative and conversion disorders), or not mapped to any ICD-10-CM code.

Figure 4B shows 14 text descriptions associated with an ICD-9-CM code of 298.9 and their ICD-10-CM mappings from site 4, where we observed the smallest decrease in the frequency of psychosis codes. Thirteen descriptors contained the word “psychosis” and 7 of these were mapped to ICD-10-CM F29. The remaining term was “confusion” and was mapped to ICD-10-CM R41.0 (Disorientation).

## Discussion

Using medical records data from ten large health systems, we find that rates of many mental health diagnosis categories remained relatively stable or increased or declined gradually during the two years before and after the transition from ICD-9-CM to ICD-10-CM, and these trends were generally similar across the ten individual health systems. Our findings were also consistent with the analysis by Yoon and Chow, who found significant but small changes in annual rates of depression, schizophrenia, and bipolar disorder between 2014 and 2016 in the Veterans Affairs (VA) health care system [18].

Uniquely, psychotic disorder diagnosis rates decreased abruptly in October 2015 by more than one third; this decrease in other psychotic disorder diagnoses was unexpected. Although schizoaffective disorder moves from a subtype of schizophrenia to its own category, and three ICD-9-CM codes for other psychosis map to a single ICD-10 code as shown in Table 1, this is a relatively straightforward crosswalk. The bulk of the change in other psychosis rates was seen in older adults and for the specific codes 298.9 and F29 (Unspecified psychosis). The impact of a single code, a phenomenon also observed in the Alcohol Use chapter [19] led us to examine the mapping of free-text labels to this code pair in a subset of source EHR systems. At two sites, the number of text descriptions with meanings other than psychosis correlated with the magnitude of the decrease in frequency of psychosis diagnosis after the ICD-10-CM transition. We hypothesize that the apparent decrease in other psychotic disorder diagnoses was due to a change in the mapping of free-text labels to diagnostic codes – with the ICD-9-CM mapping including many non-specific terms and the ICD-10-CM mapping including a narrower range of possible labels.

The decrease in rates of psychosis, specifically unspecified psychosis, is significant for early intervention efforts [12]. In our earlier work, we found, most initial presentations received less specific diagnoses, such as “unspecified psychosis” with diagnoses of schizophrenia spectrum disorder being recorded later in the course of illness. In addition, a large proportion of initial ICD-9-CM diagnoses of psychotic disorder appeared to be false positives, with no clear documentation of psychotic symptoms in full-text medical records, especially among older adults seen in general medical settings. Therefore, a possible explanation of these findings is that older adults presenting with confusion related to cognitive impairment or general medical illness received inappropriate diagnoses of unspecified psychotic disorder in the ICD-9-CM system but received more appropriate diagnoses from chapter R (symptoms, signs and ill-defined conditions) in the ICD-10-CM system.

However, data presented here were limited to diagnostic codes ascertained from health system EHRs or insurance claims. We were not able to investigate or confirm accuracy of diagnoses using actual clinical records. Neither were we able to conduct any structured examinations or other standardized assessments. Consequently, we cannot directly assess the validity or rate of false positive error for diagnoses before or after the transition to ICD-10-CM. Although chart review akin to that performed for ICD-9-CM codes [15] will be necessary to assess the confirmation rate of psychosis cases ascertained from ICD-10-CM, we expect an increased specificity of coding that will facilitate research and early intervention programs for presentation of psychotic symptoms, especially in older adults.

All sites in this study shared the same EHR software vendor, although methods of implementing the ICD-10-CM schema differed. We cannot be certain that our findings would generalize to other settings using different EHR systems. In fact, the effect of the transition to ICD-10-CM on rates of psychosis diagnoses varied considerably among the ten health systems contributing data to this analysis. The pattern of text labels for ICD-9-CM and ICD-10-CM codes we observe in these health systems may vary across EHR products or across different implementations of the same product. The most general implication of our findings is that the specific EHR algorithms used to facilitate diagnosis code selection can impact the apparent frequency of a condition, and changes in the incidence or prevalence of any diagnosis during the period from 2015 to 2017 should be carefully interpreted and further investigated.

The strengths of this study include a large sample size and the ability to investigate the functioning of the clinical user interface. In addition to offering reassurance regarding 7 of the 8 mental health conditions we analyzed and specific guidance about interpretation of changes in coding of psychotic disorders, our findings also illustrate generalizable principles for exploring anomalies or unexpected findings in data derived from health records. First, analyses stratified by health system, data source, and/or demographic groups are often illuminating. In this study, the finding of sudden decrease in diagnosis of other psychotic disorder was further explored with analyses stratified by health system and age. The marked site and age differences seen in those stratified analyses pointed to differences in coding systems with a preferential effect on older adults. Second, local knowledge of technical and organizational influences on coding or recording is invaluable. In this specific case, local examination of the mapping of text descriptors to a single diagnosis code led to the putative source of a dramatic, but artefactual, shift in diagnosis rates.

## Conclusions

The impact of the transition from ICD-9-CM to ICD-10-CM appeared to be minimal for 7 of 8 mental health diagnosis categories assessed. For the exception, psychotic disorders excluding schizophrenia and schizoaffective disorder, our findings suggest that the implementation of ICD-10-CM may have resulted in more specific use of certain codes in ten health systems, resulting in a spurious decrease in the rate of recorded diagnosis for psychosis. A more general lesson from these results is that the linkage of diagnosis codes with specific search terms in clinical terminology tools can impact clinical usage enough to cause an apparent change in prevalence. These findings support the recommendation that trend analysis and validation of diagnosis codes after the ICD-10-CM transition is necessary for all users of this clinical data.

## Additional File

The additional file for this article can be found as follows:

10.5334/egems.281.s1 Supplemental Figure 1. Monthly rates of six mental health conditions, by site.
