# Supplementary figures and images for: Impact of ICD-10-CM Transition on Mental Health Diagnoses Recording
Source: EGEMS (Wash DC). 2019 Apr 12;7(1):14. doi: 10.5334/egems.281 (PMC6484373; doi:10.5334/egems.281)

Supplemental Figure 1: Monthly rates of six mental health conditions , by site

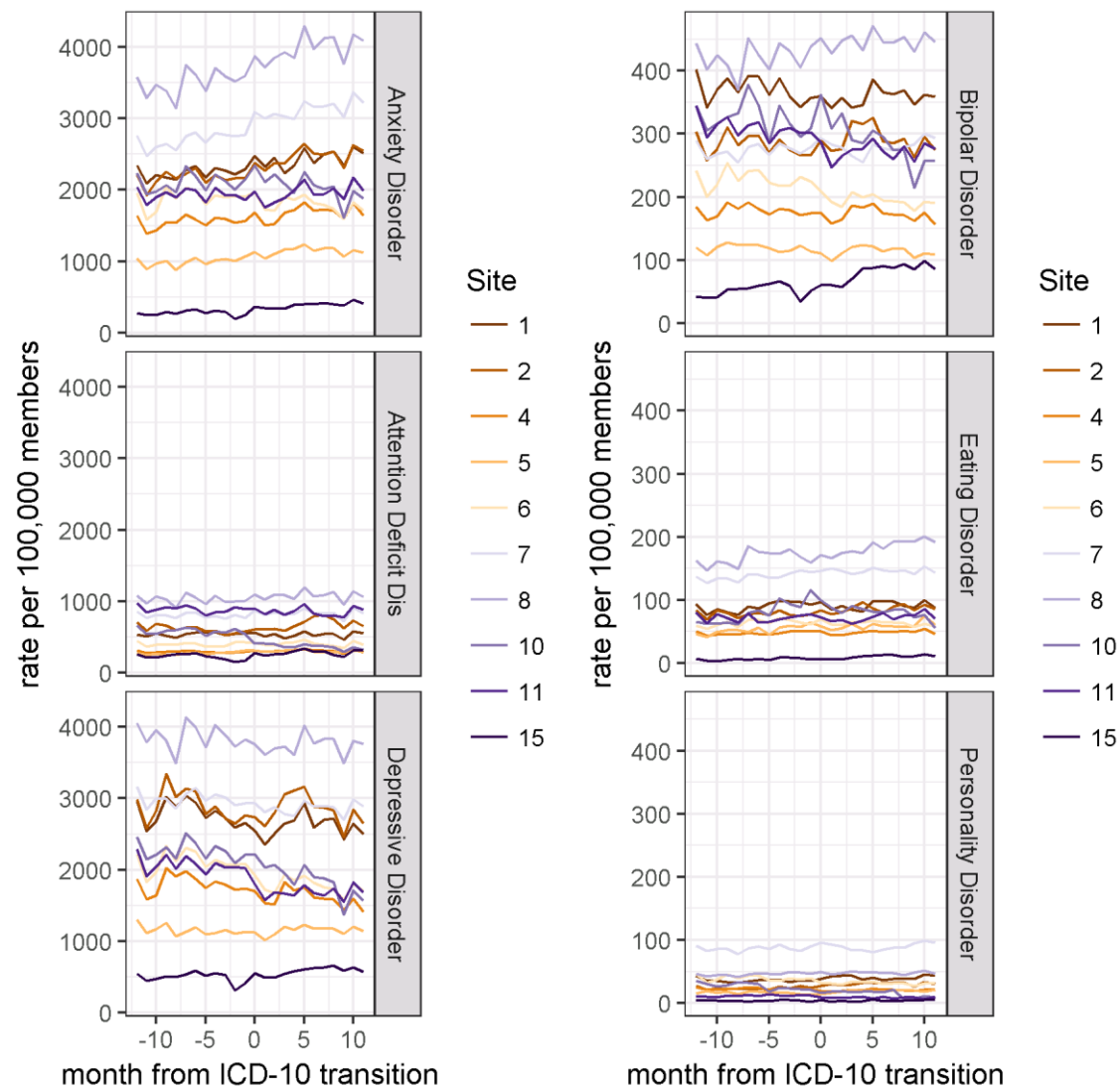

Supplement: Supplemental Figure 1. — Monthly rates of six mental health conditions, by site. [file egems-7-1-281-s1.pdf]
